# Transscleral Micropulse Cyclophotocoagulation for the Treatment of Refractory Primary Congenital Glaucoma: A Case Series

**DOI:** 10.7759/cureus.106914

**Published:** 2026-04-12

**Authors:** Shuji Hirano, Hidekazu Inami, Taiga Inooka, Ryo Tomita, Kenya Yuki

**Affiliations:** 1 Ophthalmology, Nagoya University Graduate School of Medicine, Aichi, JPN

**Keywords:** childhood glaucoma, cyclodestructive procedure, intraocular pressure (iop), laser treatment, micropulse transscleral cyclophotocoagulation, pediatric glaucoma, primary congenital glaucoma, refractory childhood glaucoma

## Abstract

Primary congenital glaucoma is a rare but vision-threatening disease. Transscleral micropulse cyclophotocoagulation (MPCPC) delivers diode laser energy in short pulses with intervening off periods, allowing tissue cooling and potentially reducing adjacent thermal damage. However, evidence on MPCPC for childhood glaucoma among Japanese patients remains limited. In this series, we describe our experience with MPCPC in refractory cases of primary congenital glaucoma.

All procedures were performed under general anesthesia. All four patients had primary congenital glaucoma that had remained uncontrolled despite multiple previous surgeries. Case 1 was a 12-year-old female who had undergone three trabeculotomies in the right eye. Her intraocular pressure (IOP) decreased transiently after MPCPC but rose again within three months. Case 2 was an 11-year-old male who had undergone three trabeculotomies in the right eye. Although the right eye showed an initial reduction in IOP after MPCPC, the effect was not sustained. Case 3 was a 10-year-old male who had previously undergone two trabeculotomies and Ahmed glaucoma valve implantation in the right eye. His IOP remained inadequately controlled after MPCPC. Case 4 was a three-year-old male who had undergone two trabeculotomies in the right eye. His IOP decreased for several months after MPCPC but rose again at nine months. No vision-threatening complications were observed. MPCPC for primary congenital glaucoma in eyes with multiple prior glaucoma surgeries may offer a favorable safety profile but limited and short-lived efficacy.

## Introduction

Primary congenital glaucoma is a rare but vision-threatening disease. In the British Infantile and Childhood Glaucoma Eye Study, the annual incidence of primary congenital glaucoma was reported as 5.4 per 100,000 live births in Great Britain and 3.3 per 100,000 live births in the Republic of Ireland [1]. More recently, a nationwide population-based study from South Korea reported a mean incidence of 1.5 per 100,000 person-years and demonstrated temporal changes in the epidemiology of childhood glaucoma in an East Asian population [2]. The primary pathophysiological mechanism of primary congenital glaucoma is increased aqueous humor outflow resistance caused by trabecular meshwork dysgenesis, leading to elevated intraocular pressure (IOP) [1-3]. Accordingly, surgical incision of the trabecular meshwork is considered an effective treatment strategy for primary congenital glaucoma [4-6]. Procedures targeting the trabecular meshwork, including ab externo trabeculotomy, ab interno trabeculotomy, and gonioscopy-assisted transluminal trabeculotomy, have shown favorable outcomes in primary congenital glaucoma [4-6].

Ikeda et al. reported IOP normalization in 86% of patients after initial ab externo trabeculotomy, and only 3.5% (4/112 eyes) required additional surgery after one to three procedures [4]. Edo et al. reported that, in a series of 20 eyes of 20 patients with Primary congenital glaucoma, ab externo trabeculotomy achieved favorable long-term outcomes, with a cumulative success rate of 89.7% at five years after the first surgery [5]. Ling et al. reported in a meta-analysis of five studies that microcatheter-assisted circumferential trabeculotomy achieved better IOP control, higher complete and qualified success rates, and reduced antiglaucoma medication use compared with conventional trabeculotomy in childhood glaucoma [6]. However, when adequate IOP control is not achieved after repeated trabeculotomies, additional surgical interventions, such as trabeculectomy, PreserFlo MicroShunt implantation, glaucoma drainage device implantation, or cyclophotocoagulation, may be required [7-10].

Transscleral micropulse cyclophotocoagulation (MPCPC) is a laser procedure used to lower IOP in patients with glaucoma [11]. Unlike conventional continuous-wave transscleral cyclophotocoagulation, MPCPC delivers a near-infrared, long-wavelength 810-nm diode laser energy in short, repetitive pulsed bursts separated by off intervals, with a duty cycle of 31.3%, allowing tissue cooling between pulses and potentially reducing collateral thermal damage while maintaining therapeutic efficacy. Therefore, MPCPC has been considered a less destructive and potentially safer alternative to traditional cyclodestructive procedures.

In this procedure, the MPCPC probe is designed to conform to the curvature of the globe and is applied firmly over the ciliary body. A small amount of viscoelastic material is placed in the fluid reservoir of the probe to improve coupling. The probe is swept circumferentially along the limbus, avoiding the three- and nine-o’clock meridians. Treatment is delivered at 2500 mW over a duration of 80 seconds per hemisphere (superior and inferior). Laser application is performed by moving the probe at a constant speed, completing one pass over a hemicircumference in approximately 20 seconds, then reversing direction and repeating the motion. This back-and-forth sweeping is repeated four times per hemisphere.

To our knowledge, there have been few reports on MPCPC for childhood glaucoma in Japanese patients. Herein, we report our clinical experience with MPCPC in primary congenital glaucoma. The objective of this study is to report the outcomes of MPCPC in Japanese patients with refractory primary congenital glaucoma.

Informed consent for publication was obtained from the parents of all children included in this study.

## Case presentation

MPCPC procedure

All procedures were performed under general anesthesia by a single surgeon (KY). The MPCPC probe was applied firmly over the ciliary body and swept circumferentially along the limbus, avoiding the three- and nine-o’clock meridians. Treatment was delivered at 2500 mW with a 31.3% duty cycle for 50 or 80 seconds per hemisphere. Preoperative antiglaucoma medications were continued postoperatively. No routine postoperative topical antibiotics, corticosteroids, or non-steroidal anti-inflammatory drugs were prescribed. All patients were hospitalized for one day postoperatively and examined by the operating surgeon. Inadequate IOP control or treatment failure was defined as an IOP ≥25 mmHg under medical therapy.

Case 1

**Figure 1:**
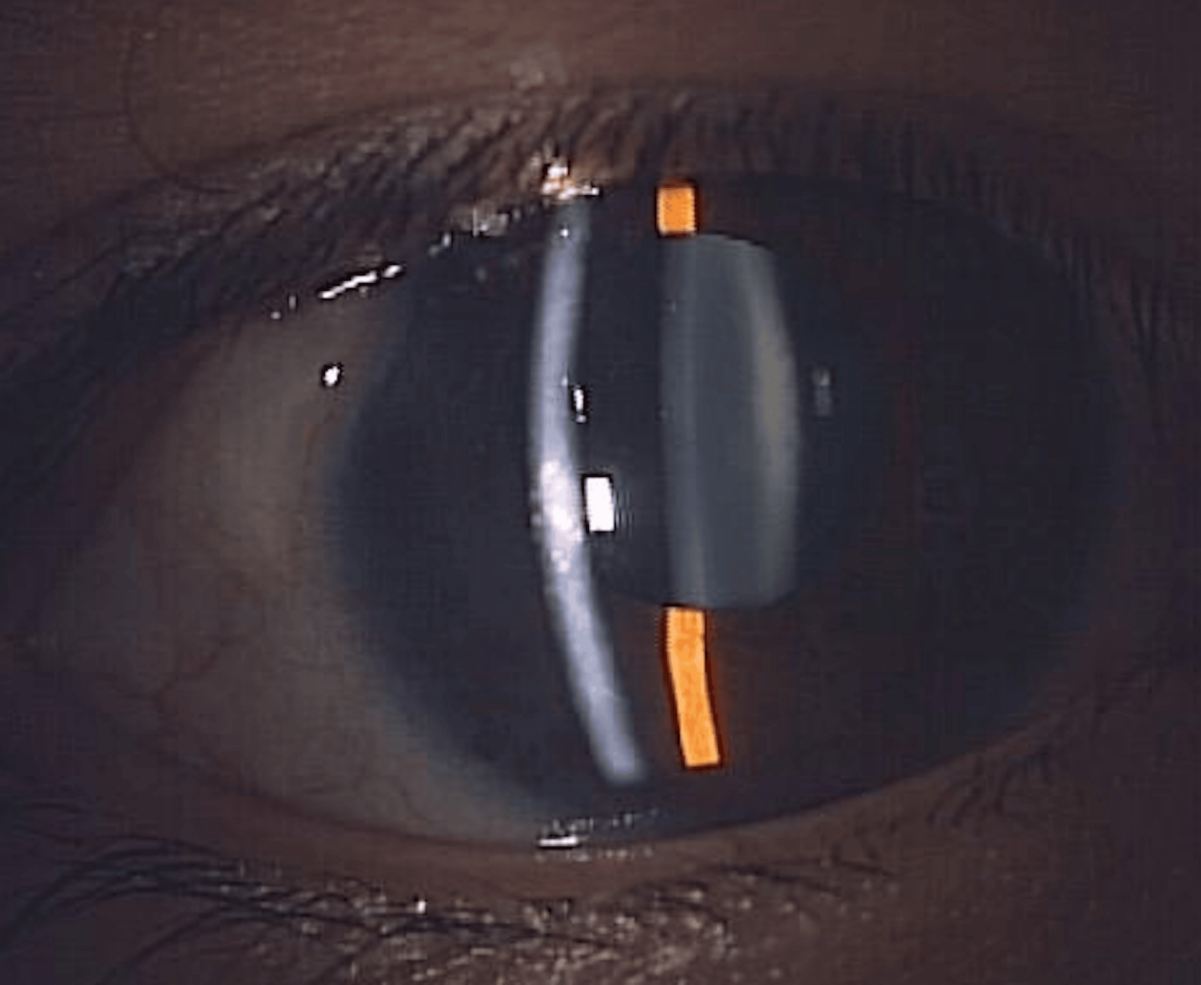
Slit-lamp microscopy of the right eye in case 1 Slit-lamp microscopy revealed no obvious abnormalities

A 12-year-old female with right primary congenital glaucoma had previously undergone three trabeculotomies in her right eye. Slit-lamp microscopy revealed no obvious abnormalities in the right eye (Figure 1).

**Figure 2:**
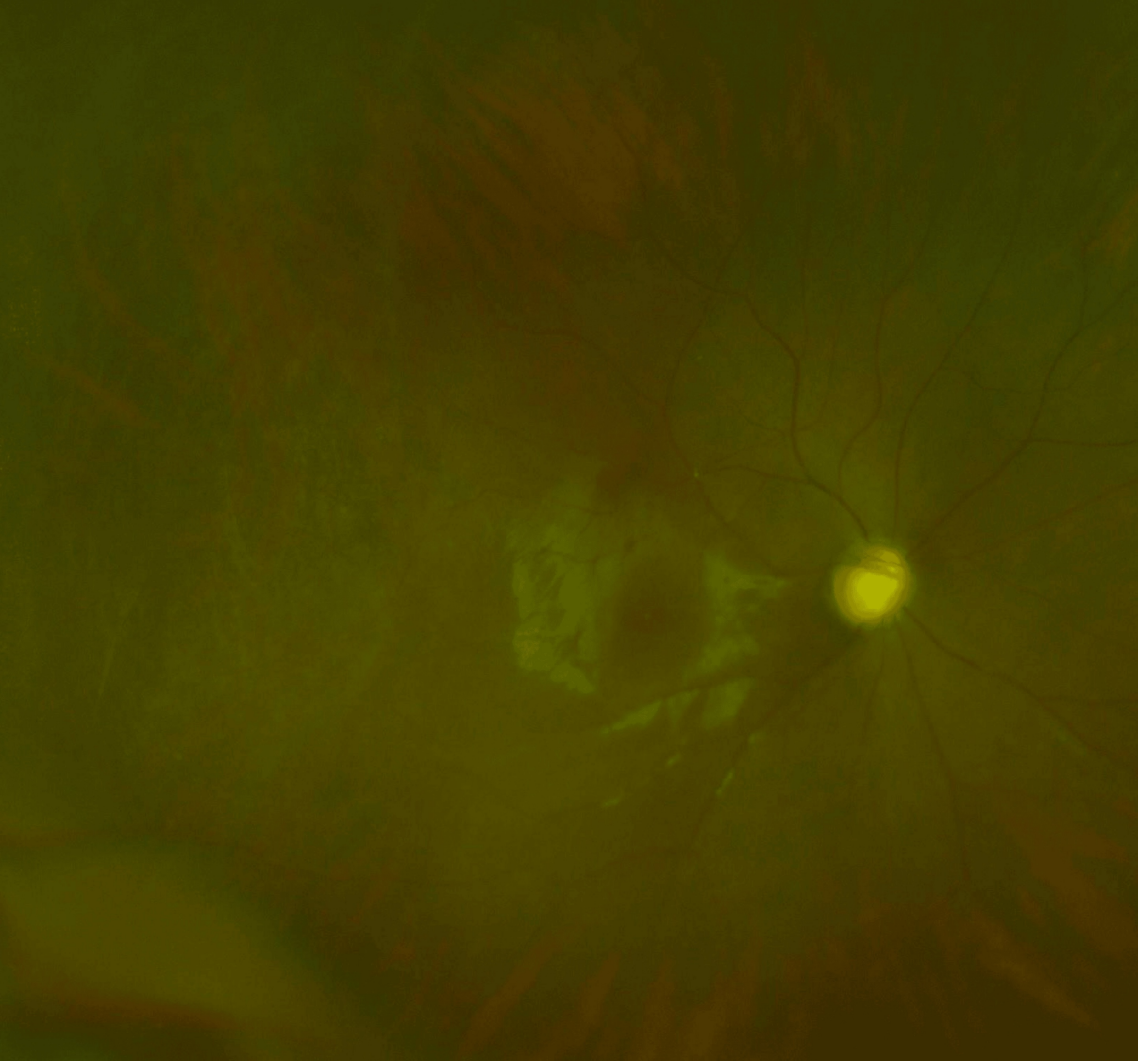
The fundus image of the right eye in case 1 The fundus of the right eye shows advanced glaucomatous optic disc cupping with a cup-to-disc ratio of 0.9

The fundus of the right eye shows advanced glaucomatous optic disc cupping with a cup-to-disc ratio of 0.9 (Figure 2).

The left eye underwent vitrectomy at two months of age for persistent fetal vasculature and has since lost light perception. Before additional intervention in the right eye, the IOP in the right eye was 24 mmHg while the patient was receiving a latanoprost/carteolol fixed-combination drug (latanoprost 0.005%/carteolol 2%) once a day, ripasudil 0.4% twice a day, and brinzolamide 1% twice a day. The patient underwent MPCPC in the right eye (2,500 mW, 50 seconds × 2). Postoperative IOP was 19 mmHg on postoperative day one and 16 mmHg at one week; however, it subsequently increased to 31 mmHg at one month and 35 mmHg at three months despite antiglaucoma medication. Given inadequate IOP control, PreserFlo MicroShunt implantation was performed in the right eye four months after MPCPC. Thereafter, IOP remained stable at approximately 7 mmHg.

Case 2

**Figure 3:**
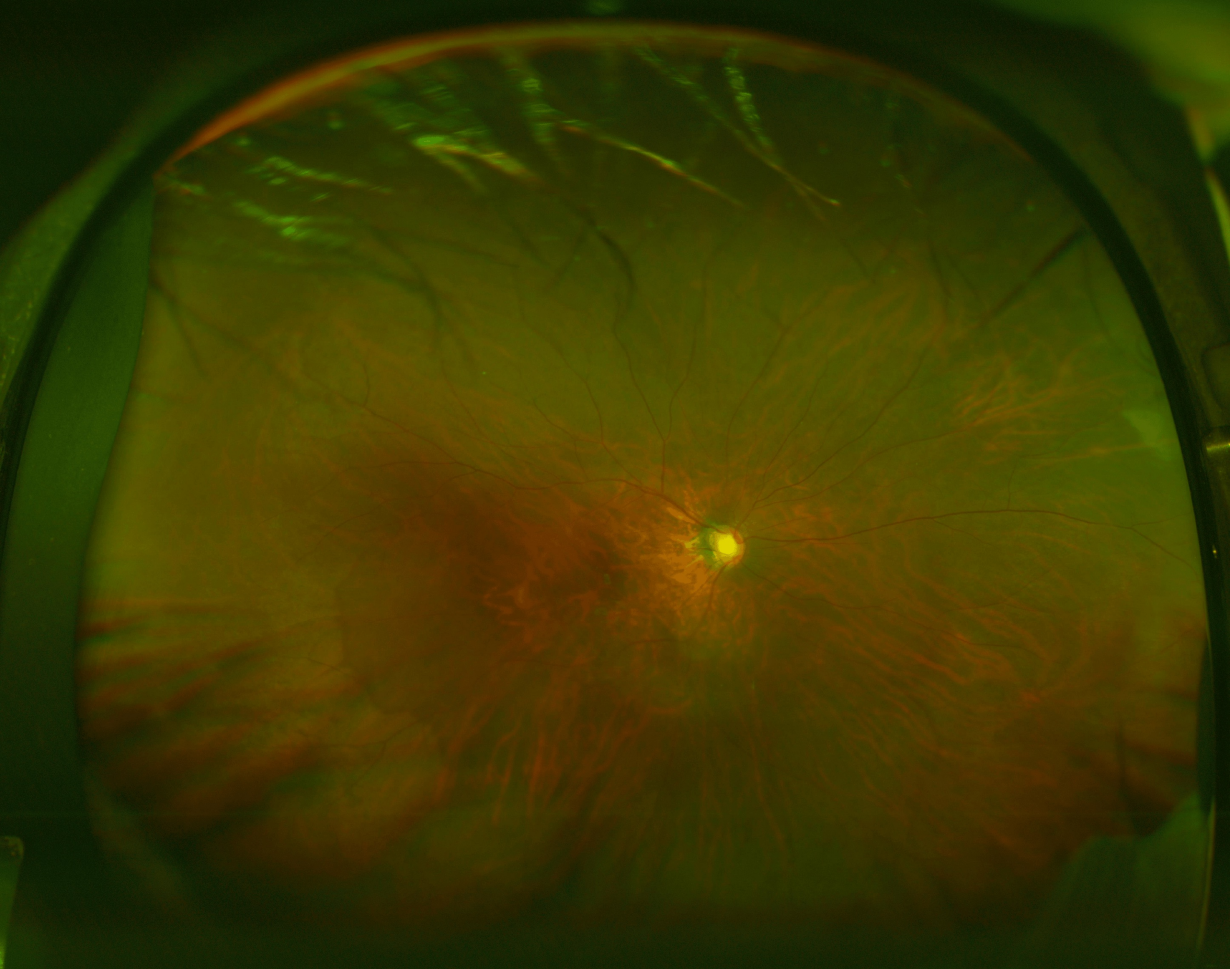
The fundus image of the right eye in case 2 The fundus of the right eye shows glaucomatous optic disc cupping with a cup-to-disc ratio of 0.8

An 11-year-old male with bilateral primary congenital glaucoma had previously undergone three trabeculotomies in the right eye. The fundus of the right eye shows advanced glaucomatous optic disc cupping with a cup-to-disc ratio of 0.8 (Figure 3).

In the left eye, a single trabeculotomy normalized the IOP, and the current best-corrected visual acuity is 0.0 logMAR. Before further intervention in the right eye, the IOP in the right eye was 33 mmHg. He underwent MPCPC in the right eye (2,500 mW, 50 seconds × 2). Postoperative IOP was 22 mmHg on postoperative day one and 11 mmHg at one week, but it increased to 34 mmHg at one month. A second MPCPC session was performed in the right eye two months later. Although IOP temporarily decreased to approximately 25 mmHg, it subsequently rose again to >30 mmHg. Given insufficient and unsustained IOP control, PreserFlo MicroShunt implantation was performed in the right eye two months later. Thereafter, IOP remained stable at approximately 10 mmHg.

Case 3

**Figure 4:**
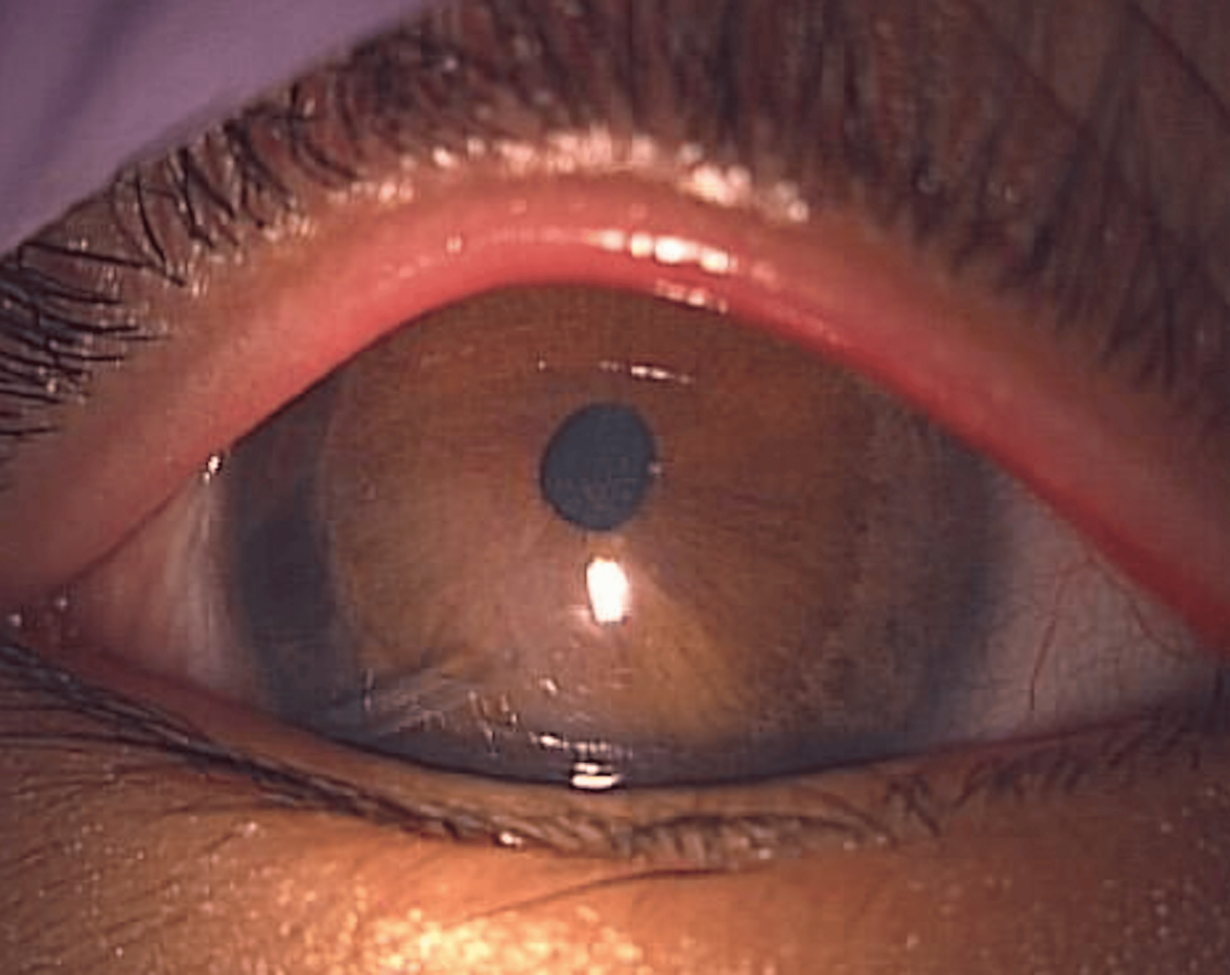
Slit-lamp microscopy of the right eye in case 3 Slit-lamp microscopy revealed an Ahmed glaucoma valve tube in the inferotemporal anterior chamber of the right eye

A 10-year-old male with bilateral primary congenital glaucoma had previously undergone two trabeculotomies and one Ahmed glaucoma valve implantation in the right eye. Slit-lamp microscopy revealed an Ahmed glaucoma valve tube in the inferotemporal anterior chamber of the right eye (Figure 4).

**Figure 5:**
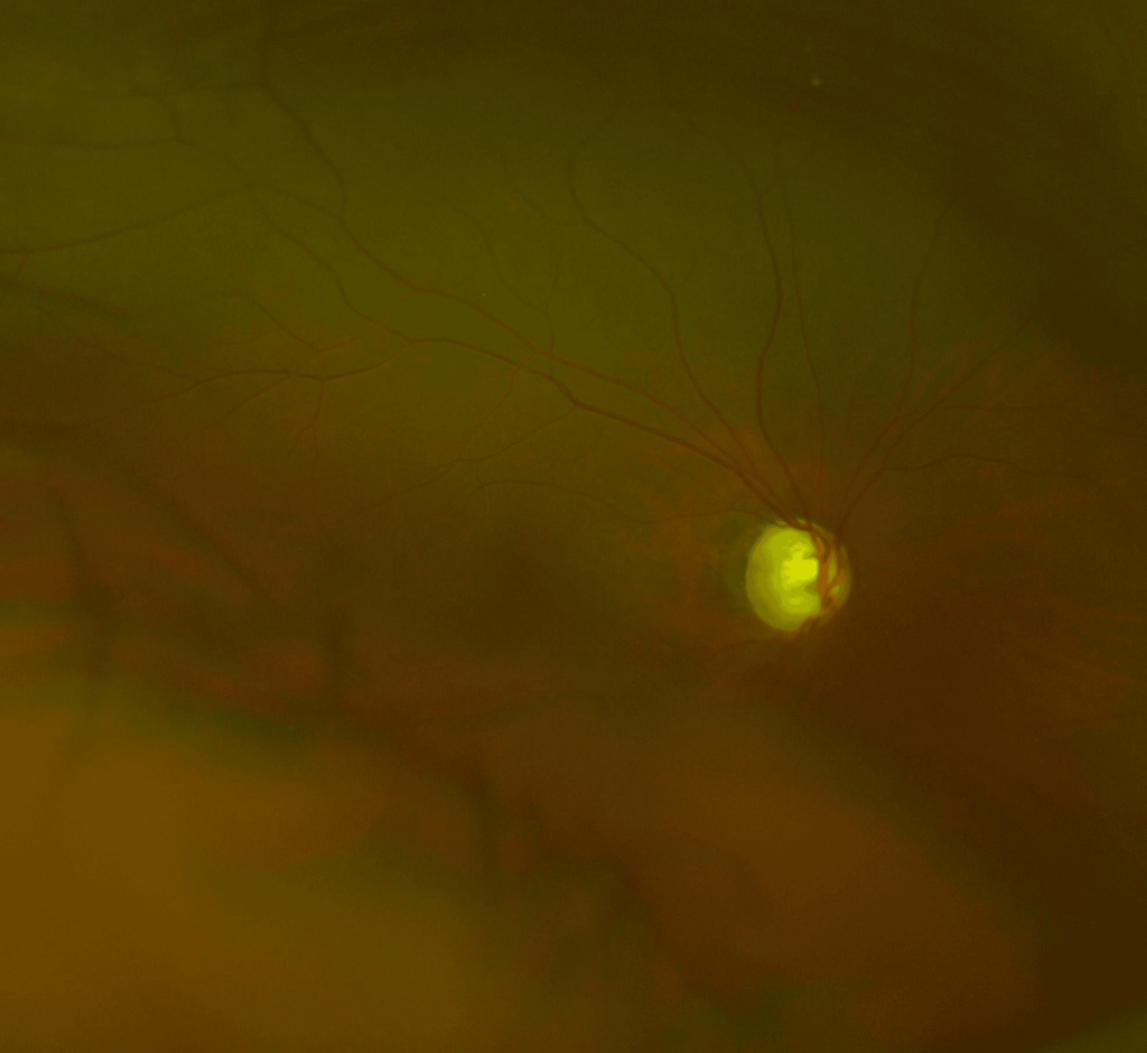
The fundus image of the right eye in case 3 The fundus image of the right eye shows advanced glaucomatous optic disc cupping with a cup-to-disc ratio of 1.0

The fundus of the right eye shows advanced glaucomatous optic disc cupping with a cup-to-disc ratio of 1.0 (Figure 5).

The left eye had previously undergone two trabeculotomies, one Ahmed glaucoma valve implantation, one Baerveldt glaucoma implant surgery, and one PreserFlo MicroShunt implantation. IOP is currently controlled, and the best-corrected visual acuity is 2.0 logMAR. Before further intervention in the right eye, the IOP in the right eye was 29 mmHg while the patient was receiving a latanoprost/carteolol fixed-combination drug (latanoprost 0.005%/carteolol 2%) once a day, ripasudil 0.4% twice a day, and brinzolamide 1% twice a day. He underwent MPCPC in the right eye (2,500 mW, 80 seconds × 2). Postoperative IOP was 21 mmHg on postoperative day one, but it increased to 32 mmHg at one week and 34 mmHg at one month. Given insufficient IOP control, Baerveldt glaucoma implant surgery was performed in the right eye three months after MPCPC. Thereafter, IOP decreased to the high-teen range. 

Case 4

**Figure 6:**
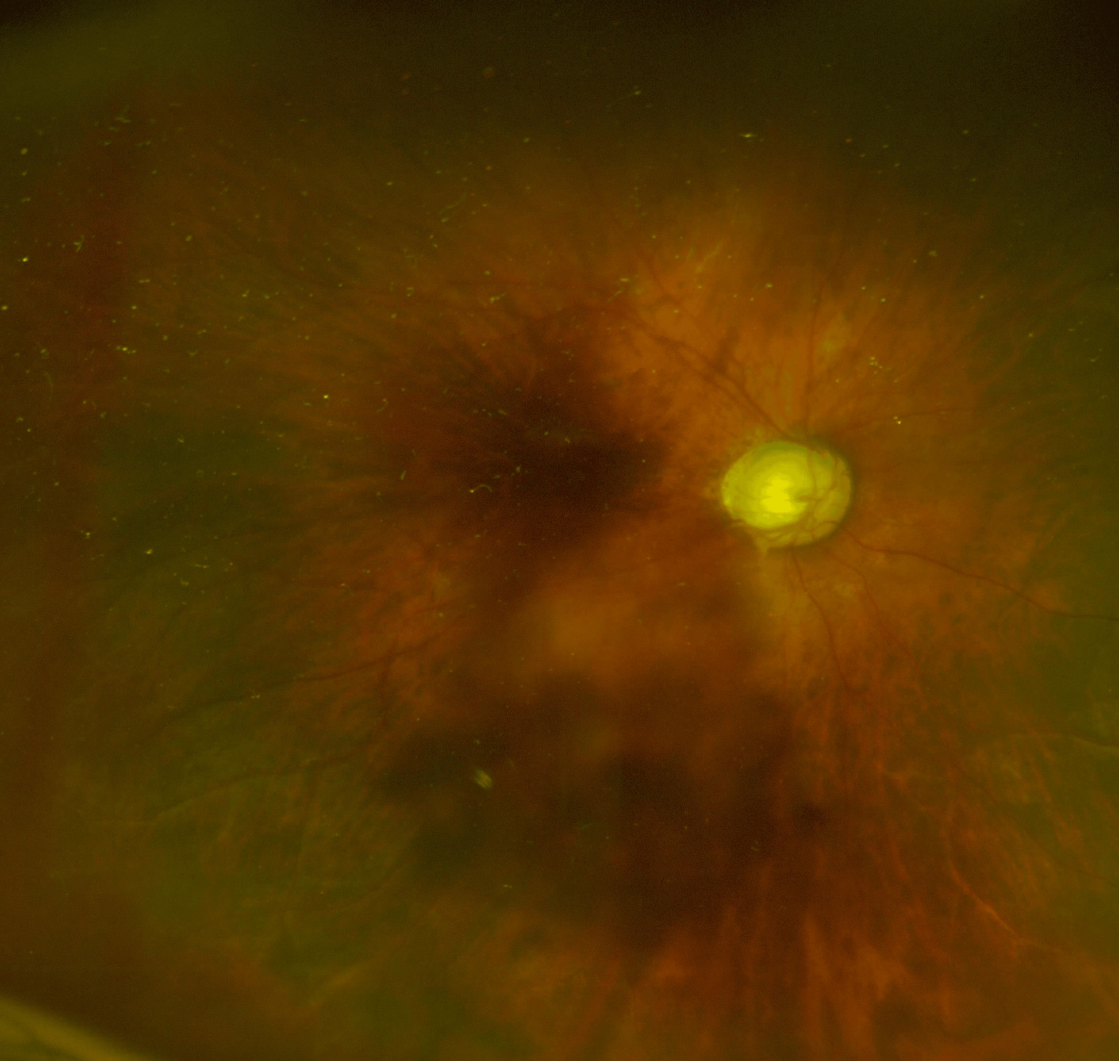
The fundus image of the right eye in case 4 The fundus of the right eye shows glaucomatous optic disc cupping with a cup-to-disc ratio of 0.9

A 3-year-old male with right primary congenital glaucoma had previously undergone two trabeculotomies. The fundus of the right eye shows glaucomatous optic disc cupping with a cup-to-disc ratio of 0.9 (Figure 6).

The left eye achieved normal IOP after two trabeculotomies, and the best-corrected visual acuity is 0.82 logMAR. Before additional intervention in the right eye, the IOP in the right eye was 42 mmHg. He underwent MPCPC in the right eye (2,500 mW, 50 seconds × 2). Postoperative IOP decreased to 29 mmHg on postoperative day one, 28 mmHg at one week, 25 mmHg at one month, and 21 mmHg at three months; however, it increased again to 38 mmHg at nine months. Given the rebound elevation of IOP, PreserFlo MicroShunt implantation was performed in the right eye nine months after MPCPC. Thereafter, IOP remained around 20 mmHg.

In our series, three eyes showed inadequate IOP control within one month after MPCPC, whereas one eye developed inadequate IOP control at nine months. Consequently, additional filtering surgery was required in all cases. No vision-threatening complications were observed. In particular, there were no cases of choroidal detachment, marked hypotony, or corneal ulceration.

A summary of all four cases is presented in Table 1.

**Table 1:** Summary of patient characteristics, prior surgeries, IOP outcomes, and final interventions The median number of prior glaucoma surgeries per eye before MPCPC was 3 (range: 2-3). IOP: intraocular pressure; LOT: trabeculotomy; MPCPC: micropulse cyclophotocoagulation; 1D: postoperative day 1; 1W: 1 week postoperatively; 1M: 1 month postoperatively; 3M: 3 months postoperatively; PCG: primary congenital glaucoma; AGV: Ahmed glaucoma valve; Microshunt: PreserFlo MicroShunt; BGI: Baerveldt glaucoma implant

Case	Age (years)	Sex	Type of glaucoma	LOT history (times)	Glaucoma surgery history	MPCPC parameters	Preoperative IOP (mmHg)	1D IOP (mmHg)	1W IOP (mmHg)	1M IOP (mmHg)	3M IOP (mmHg)	IOP before additional glaucoma surgery (mmHg)	Time to additional glaucoma surgery (months)	Final intervention
1	12	Female	PCG	3	-	2500mW 50sec	24	19	16	31	35	35	4	Microshunt
2	11	Male	PCG	3	-	2500mW 50sec	33	22	11	34	40	30	4	Microshunt
3	10	Male	PCG	2	AGV	2500mW 80sec	29	21	32	34	-	34	1	BGI
4	3	Male	PCG	2	-	2500mW 50sec	42	28	28	25	21	38	9	Microshunt

## Discussion

We investigated the surgical outcomes of MPCPC for primary congenital glaucoma. In our series, three eyes showed inadequate IOP control within one month after MPCPC, and the remaining eye failed at nine months. Consequently, all eyes required additional glaucoma surgery.

Balbaid et al. evaluated the efficacy and safety of MPCPC in pediatric glaucoma in a retrospective case series of 44 eyes (patients aged <17 years) with at least one year of follow-up. Approximately 60% of eyes had primary congenital glaucoma, and the mean age was 10 years [12]. Most eyes (79.5%) had undergone prior glaucoma surgery, and more than half had a history of tube-shunt implantation. Mean IOP decreased significantly from 32.7 ± 8.7 mmHg at baseline to 23.2 ± 8.6 mmHg at six months and 21.7 ± 7.9 mmHg at one year (p < 0.0001). Using IOP normalization with or without antiglaucoma medications as the definition of success, the success rate was 47.5% at six months and 53.5% at one year. Hypotony occurred in two eyes, and one eye developed a neurotrophic corneal ulcer.

Sivasubramanian et al. evaluated MPCPC outcomes in refractory pediatric glaucoma in a prospective interventional study of 23 eyes of 23 patients (age <18 years) with six months of follow-up [13]. The mean age was 11 years; 25% had primary congenital glaucoma and 25% had glaucoma following vitrectomy. Approximately 80% had a history of prior glaucoma surgery, and half of those had undergone trabeculectomy. IOP decreased significantly at all follow-up visits compared with baseline (p = 0.00001). However, additional interventions were required in 8.6% of eyes within one month, 26% between one and three months, and 13% between three and six months. Overall success, defined as IOP normalization, was 56.5% at one month and 34.7% at six months. No major complications were reported.

Elhefney et al. conducted a prospective study of micropulsed diode laser cyclophotocoagulation in children (age <16 years) with pediatric glaucoma (36 eyes, 29 patients; median age 24 months) with a mean follow-up of approximately 15 months [14]. Mean IOP decreased significantly from 37.5 ± 11.3 mmHg to 20.0 ± 2.7 mmHg at 15 months (p < 0.001), and the mean number of glaucoma medications decreased from 2.6 ± 0.5 to 1.7 ± 0.6 (p < 0.001). Repeat treatment was common (66.7%), with a mean of 1.7 ± 0.5 sessions per eye. The cumulative probability of qualified success at 12 months was 47%, and no major ocular complications were reported.

Elhusseiny et al. evaluated the outcomes of continuous wave transscleral cyclophotocoagulation (CWCPC) in childhood glaucoma in a meta-analysis of 17 studies comprising 526 patients (658 eyes) [15]. In the CWCPC group, the median follow-up was 28 months, and the mean number of TS-CPC sessions was 1.7, indicating that repeat treatment was common. Mean IOP decreased significantly from 31.2 ± 8 mmHg preoperatively to 20.8 ± 8 mmHg at the last follow-up (p < 0.001), whereas the mean number of glaucoma medications did not change significantly (2.3 ± 1.3 to 2.2 ± 1.3; p = 0.37). Among studies reporting complications (10 studies, 324 eyes), adverse events included conjunctival hyperemia (4%), anterior chamber reaction (2%), phthisis bulbi (2%), retinal detachment (1%), and progression to no light perception (1%). In addition, 10% of eyes subsequently required glaucoma drainage device implantation. Overall, complications were observed in 19% (60/324) of CWCPC-treated eyes.

Wang et al. compared MPCPC with continuous-wave CWCPC in a retrospective study, defining success as IOP normalization on any number of topical medications [16]. In the MPCPC group, the mean age was seven years, and 40% had primary congenital glaucoma. Among 48 patients, 26 eyes underwent MPCPC, and 30 eyes underwent CWCPC. At one year, success was achieved in 26.9% (7/26 eyes) in the MPCPC group and 43.3% (13/30 eyes) in the CWCPC group; survival analysis showed a significant difference favoring CWCPC (p = 0.03). One eye developed persistent hypotony in the CWCPC group, whereas no complications were reported in the MPCPC group.

Abdelrahman et al., in a prospective study of 45 eyes of 36 children with refractory glaucoma, found that MPCPC and CWCPC achieved comparable IOP reduction, with no significant difference between the two groups (63% vs. 67%, p = 0.6), indicating similar short-term efficacy in lowering IOP in this pediatric population [17]. Although the difference was not statistically significant, the MPCPC group showed a trend toward fewer complications, as no significant complications were observed, whereas one eye in the CWCPC group developed phthisis bulbi and two eyes had severe pain and uveitis. Vega-Garces et al. also conducted a retrospective cohort study comparing MPCPC and CWCPC in pediatric glaucoma (28 patients, 81 eyes) [18]. Although both treatments lowered IOP in the short term, CWCPC showed a larger mean IOP decrease than MPCPC at 12 months (7.99 ± 7.95 vs. 1.78 ± 6.89 mmHg, p < 0.05), whereas the difference in success rates (27% vs. 22%) was not statistically significant.

Overall, the reported efficacy of MPCPC in pediatric glaucoma appears modest, and the need for repeat treatment or additional interventions is common. However, direct comparisons across studies should be interpreted cautiously because success definitions, baseline severity, and treatment parameters varied among reports. In our study, all eyes showed early failure (within two months), and our outcomes were poorer than those previously reported. This discrepancy may reflect greater disease severity in our cohort. In our study, the median number of prior glaucoma surgeries per eye before MPCPC was three (range: two to three). The mean preoperative IOP was 32.0 mmHg (24, 33, 29, and 42 mmHg). A prior glaucoma drainage device had been implanted in one of four eyes (25%) before MPCPC (Ahmed glaucoma valve). Given the markedly severe and highly refractory nature of our cases, it is possible that our outcomes were poorer than those reported in previous studies. 

Although cyclodestructive procedures can be associated with vision-threatening complications, including inflammation, hypotony, choroidal detachment, macular edema, sympathetic ophthalmia, and phthisis bulbi [10]. MPCPC has generally been reported to have a favorable safety profile in pediatric glaucoma. Across the above studies, complications were uncommon: Balbaid et al. reported two cases of hypotony and one neurotrophic corneal ulcer [12], whereas the other studies reported no major complications. Similarly, we observed no apparent complications attributable to MPCPC in our series. In contrast, CWCPC has been associated with complications such as persistent hypotony in pediatric cases; therefore, MPCPC may offer a more favorable safety profile than CWCPC.

## Conclusions

MPCPC for primary congenital glaucoma, particularly in eyes with multiple prior glaucoma surgeries, may offer a favorable safety profile but limited and short-lived efficacy. In such highly refractory cases, alternative surgical strategies, including filtering procedures (e.g., glaucoma drainage devices or microshunt implantation) or CWCPC, should be considered.
